# Cognitive Diagnosis Computerized Adaptive Testing (CD-CAT) for Adolescent Internet Gaming Disorder: A Conceptual Assessment Framework

**DOI:** 10.3390/bs16040558

**Published:** 2026-04-08

**Authors:** Min Jia, Jing Liu

**Affiliations:** 1 Key Laboratory of the Ministry of Education on Adolescent Cyber Psychology and Behavior, Central China Normal University, Wuhan 430079, China; 2 School of Psychology, Central China Normal University, Wuhan 430079, China

**Keywords:** adolescents, internet gaming disorder, cognitive diagnosis computerized adaptive testing, conceptual assessment framework

## Abstract

Internet Gaming Disorder (IGD) has become a major behavioral health concern among adolescents, yet current assessment tools remain limited. These tools often fail to capture the disorder’s complex symptom variations and lack clinical interpretability. This study, taking an interdisciplinary approach that combines clinical psychology and psychometrics, summarizes recent progress in understanding adolescent IGD and the development of its assessment methods. We compare the diagnostic criteria of the DSM-5 TR and ICD-11 and argue that the nine DSM-5 TR criteria are particularly suited for transformation into distinct diagnostic attributes due to their detailed and actionable nature. We then review the strengths and weaknesses of Classical Test Theory (CTT), Item Response Theory (IRT), and Cognitive Diagnostic Models (CDMs) in assessing IGD. The review emphasizes the limitations of total-score and single latent-trait approaches in capturing the disorder’s multidimensional symptoms. Based on these insights, we propose a conceptual assessment framework, Cognitive Diagnosis Computerized Adaptive Testing (CD-CAT), that integrates CDMs with computerized adaptive testing. Rather than presenting an empirically validated system, this framework offers a theoretically grounded proposal that specifies the key components, logical relationships, and methodological pathways necessary for advancing precision assessment of adolescent IGD. CD-CAT uses a system of attributes and a Q-matrix based on the DSM-5 TR criteria to efficiently classify IGD symptoms in adolescents, reducing the number of items required while enhancing clinical relevance. Lastly, we discuss the theoretical contributions of the proposed framework, acknowledge its limitations as a conceptual proposal, and outline directions for future empirical research.

## 1. Introduction

With the rapid development of digital technology and the widespread use of smart devices, online gaming has become an integral part of adolescents’ daily lives worldwide. It plays a significant role in their entertainment, social interactions, and self-identity construction (Cudo et al., 2024). For a subset of adolescents, however, gaming leads to problematic patterns of behavior characterized by loss of control, functional impairment, and compulsive continued use. These behaviors are commonly referred to as Internet Gaming Disorder (IGD) (American Psychiatric Association, 2022; Paulus et al., 2018). Epidemiological studies show that the prevalence of adolescent IGD is moderate to high, with significant variation across regions (Fam, 2018; Mihara & Higuchi, 2017; Satapathy et al., 2025). This issue persists into young adulthood, indicating that IGD may continue to affect individuals over time (Gisbert-Pérez et al., 2025). Furthermore, IGD is strongly linked to negative outcomes, including lower academic performance, impaired social functioning, and psychological distress, such as anxiety and depression (Düll et al., 2024; X. Dong et al., 2022; Gentile et al., 2017; Tse et al., 2025). These findings highlight IGD as a significant public health concern and emphasize the need for accurate scientific assessment.

Despite the growing body of research on the epidemiology of IGD and its effects on adolescent mental and physical health, significant challenges remain in the assessment tools and methods. First, while the diagnostic criteria for IGD are included in both the Diagnostic and Statistical Manual of Mental Disorders, Fifth Edition (DSM-5 TR) and the International Classification of Diseases, Eleventh Revision (ICD-11), existing assessment instruments vary in structure and validity. This variation limits the comparability and consistency of findings across different studies (American Psychiatric Association, 2022; Borges et al., 2021; World Health Organization, 2019). Second, many traditional self-report scales are still based on Classical Test Theory (CTT), which calculates scores by summing symptom items under the assumption that IGD is a single continuous dimension. This approach fails to capture the diversity and complexity of symptoms, restricting the accurate identification of individualized symptom profiles in adolescents (Hu, 2022; Kökönyei et al., 2019). Even with the partial introduction of Item Response Theory (IRT) to enhance measurement precision, current tools still struggle to adequately capture IGD’s multidimensional symptom structure and individual differences (Thomas, 2019).

To address these challenges, IGD assessment requires an innovative approach that can effectively capture symptom heterogeneity while maintaining both efficiency and practical feasibility. In this regard, Cognitive Diagnostic Models (CDMs) provide a new perspective by breaking down complex constructs into discrete, clinically relevant symptom attributes, which helps uncover the internal heterogeneity of IGD. At the same time, Computerized Adaptive Testing (CAT) enhances efficiency and accuracy through dynamic item selection. By combining these two approaches into Cognitive Diagnosis Computerized Adaptive Testing (CD-CAT), it is possible to achieve a better balance between precision, efficiency, and clinical interpretability.

Therefore, this study aims to systematically summarize advancements in the conceptualization and assessment of adolescent IGD. Based on this, it proposes a conceptual assessment framework, Cognitive Diagnosis Computerized Adaptive Testing (CD-CAT), to offer a new methodological perspective for adolescent IGD assessment. Developing a well-grounded conceptual assessment framework represents a necessary and foundational contribution to the field, as it establishes the theoretical rationale, defines the core components, and specifies the logical relationships that must precede any empirical implementation.

## 2. The Construct, Heterogeneity, and Assessment Needs of Internet Gaming Disorder

### 2.1. Evolution of Diagnostic Frameworks: From DSM-5 to ICD-11

Accurately defining the clinical boundaries of IGD is essential for developing effective assessment tools. In the DSM-5 TR, IGD is included in Section 3 (“Conditions for Further Study”), officially recognizing its potential pathological status. The DSM-5 TR outlines nine diagnostic criteria, covering cognitive aspects (e.g., preoccupation with gaming, tolerance), behavioral aspects (e.g., loss of control, continued gaming despite problems), and emotional aspects (e.g., withdrawal, escape) (American Psychiatric Association, 2022). To receive a provisional IGD diagnosis, individuals must meet at least five of these nine criteria within the past 12 months. Notably, the DSM-5 TR focuses primarily on internet gaming, highlighting the role of online connectivity in the development of addictive behaviors (American Psychiatric Association, 2022; Müller et al., 2019).

In contrast, the ICD-11 took a significant step by formally including Gaming Disorder (GD) in the mental disorders section (World Health Organization, 2019). The ICD-11 definition is broader, encompassing both online and offline gaming. Its core features are summarized across three dimensions: (1) impaired control over gaming, (2) increasing priority given to gaming over other activities and interests, and (3) continued or escalating gaming despite negative consequences (Zhong et al., 2018). The ICD-11 diagnostic threshold focuses on the severity of functional impairment rather than simply counting symptoms. While DSM-5 TR and ICD-11 differ in scope and wording, both are clinically valuable for identifying pathological gaming behaviors (Borges et al., 2021).

As outlined in Table 1, the DSM-5 TR specifies nine discrete diagnostic criteria, whereas the ICD-11 has three core features related to impaired control, prioritization, and continued gaming despite negative consequences (Zhong et al., 2018). From a cognitive diagnosis perspective, the nine DSM-5 TR criteria offer a finer structure that is more suitable for being broken down into discrete, measurable attributes. Several considerations support this choice. First, the DSM-5 TR framework has accumulated a substantially larger body of validated psychometric instruments since 2013, providing an established item pool for CD-CAT item bank construction, whereas ICD-11-based tools remain limited (Pontes & Griffiths, 2015; Zhang et al., 2022). Second, item response theory analyses have confirmed that all nine DSM-5 TR criteria exhibit high discrimination parameters and substantial diagnostic information (Schivinski et al., 2018), which is essential for Q-matrix construction. Third, the three ICD-11 core features, while clinically meaningful, are defined at a level of abstraction that poses operationalization challenges for discrete binary attribute definition. It is acknowledged that certain DSM-5 TR criteria show variable diagnostic utility in adolescent samples, for instance, “tolerance” and “escape” have demonstrated weaker diagnostic contribution compared to criteria such as “loss of interest” and “continued use despite problems” (Luo et al., 2022; Rehbein et al., 2015). However, this variability itself underscores the value of a CDM-based approach: rather than summing all criteria into a single total score, CD-CAT evaluates each attribute individually, allowing differential weighting to emerge naturally from the diagnostic profile. For these reasons, the DSM-5 TR criteria serve as the theoretical basis for attribute specification in this study.

### 2.2. Symptom Heterogeneity and Deeper Assessment Needs

The clinical presentation of IGD is highly diverse and complex, often referred to as symptom heterogeneity. However, traditional total-score models tend to obscure this heterogeneity. Studies using Latent Profile Analysis (LPA) have shown that there are distinct subtypes within the IGD population. For example, Zhang et al. (2023), in a study of nearly 6000 adolescents, identified four subtypes: healthy gamers, an impulsivity-control subtype, a gaming-prioritization subtype, and a typical gaming disorder group. These subtypes reflect differences in emotion regulation, impulse control, and gaming prioritization. Similarly, Ahmed et al. (2024), in a study of 1005 adolescents, identified three subtypes: regular gamers, moderate gamers, and potentially addicted gamers, with the high-risk group showing significantly higher levels of depression, anxiety, and poorer social adjustment. Together, these findings suggest that IGD varies not only in severity but also in underlying pathological mechanisms, reflecting multidimensional heterogeneity that total-score approaches cannot capture.

Mapping these empirical findings onto the DSM-5 TR diagnostic criteria reveals three clinically meaningful subtype patterns. First, an emotion-regulation subtype, in which gaming functions primarily to relieve negative affect such as anxiety and depression, corresponding most directly to the criteria of “escape” and “withdrawal”. Second, an impulse-control subtype, in which individuals struggle to regulate gaming urges and continue gaming despite academic or social consequences, corresponding to “loss of control,” “deception,” and “continued gaming despite problems.” Third, a social/reward subtype, driven by achievement motivation and social engagement in gaming contexts, corresponding to “preoccupation” and “tolerance.”

This subtype-level variation has direct implications for clinical intervention. Interventions are typically designed based on the presence or absence of specific symptoms rather than a vague total score—for example, detoxification-like management is considered only when withdrawal symptoms are present. Emotion-regulation subtypes may benefit more from emotion management and coping skills training, while impulse-control subtypes may require impulse inhibition training and environmental adjustments (Kökönyei et al., 2019). Notably, different DSM-5 TR criteria contribute differently to predicting distress: “withdrawal” and “escape” are strong predictors, while “tolerance” and “loss of control” are comparatively weaker (Luo et al., 2022). This differential diagnostic contribution across criteria further underscores the inadequacy of equal-weighted total scoring, and reinforces the case for an attribute-level assessment approach that supports cognitive diagnosis.

### 2.3. Methodological Limitations of Existing Static Assessment Tools

Most widely used IGD measures (e.g., IGDS9-SF, GAS) are static, fixed-form instruments (Paschke et al., 2020). These tools have three main limitations: (1) informational ambiguity: a total score provides a broad indication of the condition’s severity but does not specify where the problem lies (Razum & Glavak-Tkalić, 2025). (2) inefficiency: to ensure adequate precision across respondents with varying severity levels, static scales typically include many items. For low-severity respondents, items related to severe symptoms provide little useful information, while for high-severity respondents, basic items add minimal additional value. (3) bias from dimensional assumptions: many scales assume that IGD is unidimensional, meaning that all items measure the same underlying trait. However, factor-analytic evidence suggests that IGD may have a multidimensional structure (Király et al., 2017; Poon et al., 2021). Forcing a multidimensional construct into a unidimensional model can undermine structural validity. Furthermore, inconsistencies across different measures can create clinical confusion. For instance, IGDS9-SF may show diagnostic inconsistencies when compared with other multidimensional tools (Sánchez-Iglesias et al., 2020).

## 3. Measurement Paradigms in IGD Assessment: From CTT and IRT to Cognitive Diagnostic Models

### 3.1. The Limitations of Classical Test Theory in Adolescent IGD Assessment

Classical Test Theory (CTT) has been a foundational framework in psychometrics, but its limitations become more pronounced when used to assess adolescents with highly heterogeneous presentations of Internet Gaming Disorder (IGD). The core CTT equation, X = T + E, assumes that measurement error (E) is random and consistent across individuals (Allen & Yen, 2001). However, in IGD assessment, errors tend to be systematic rather than random.

First, total scores obscure clinically meaningful differences attributable to symptom heterogeneity. For example, a subtype focused on emotion regulation (i.e., using gaming to escape negative emotions) and an impulse-control subtype may receive the same total score. Under CTT, they appear to be similar, but their underlying mechanisms differ greatly. CTT cannot distinguish these qualitative differences, leading to potential clinical misinterpretations and hindering the application of subtype-specific interventions.

Second, CTT’s reliance on sample-specific data prevents comparability across different contexts. The prevalence and severity of IGD vary significantly across different samples (e.g., general school students vs. clinical patients). An item that is considered “high difficulty” for school students may be deemed “low difficulty” for clinical patients, making it challenging to compare CTT-based scales across different settings. This limits the effectiveness of early-warning systems (Thompson, 2023).

Third, CTT fails to recognize clinical thresholds in a true interval scale. In CTT, raw scores are ordinal, meaning that the position of diagnostic thresholds (e.g., meeting five DSM-5 criteria) on the score scale is unclear. This increases the risk of both underdiagnosis and overdiagnosis (Jabrayilov et al., 2016).

### 3.2. Item Response Theory (IRT): Focus on Individual Items

Item Response Theory (IRT) employs nonlinear mathematical models to shift the focus of analysis from total test scores to individual items. One of the main advantages of IRT is parameter invariance: item parameters (e.g., difficulty b, discrimination a, and guessing c) remain consistent across different samples (Embretson & Reise, 2013), which supports the use of large-scale item banks across diverse groups and cultures. Moreover, IRT introduces the Item Information Function (IIF) and Test Information Function (TIF), which allow for precise measurement of how much information a test provides at each point on the latent trait continuum (θ). This forms the mathematical foundation for Computerized Adaptive Testing (CAT) (L. Cai et al., 2016).

However, IRT has a fundamental limitation in clinical IGD assessment: it primarily measures severity rather than categories. The continuous latent score (θ) differentiates between mild and severe cases, which is useful for epidemiological classification. However, clinicians typically do not need to identify the overall severity of the condition, but the specific symptom dimensions that are present. For example, in clinical decision-making, if withdrawal is present, the treatment might involve physiological management; if “loss of control” is present, the intervention might involve impulse-control training as part of Cognitive Behavioral Therapy (CBT). Since IRT’s θ is a composite score, it cannot directly address these decision points. Additionally, IGD symptoms are often blurred, and while IRT’s continuous scale captures these gray areas, it lacks the discrete classification thresholds that Cognitive Diagnostic Models (CDMs) provide. As a result, IRT may struggle to offer a clear “yes/no” diagnostic conclusion. In other words, while IRT provides more precise severity estimates than CTT, it remains anchored to a single latent dimension and cannot decompose IGD into the discrete clinical components that inform targeted intervention, creating a gap between assessment outcomes and intervention planning (Lim & Bangeranye, 2024).

### 3.3. Cognitive Diagnostic Models (CDMs): From Quantification to Diagnosis

Cognitive Diagnostic Models (CDMs) represent a shift from measuring continuous severity to diagnosing discrete structures. Rather than estimating an individual’s position on a single latent trait, CDMs classify whether individuals have mastered a set of predefined psychologically or clinically meaningful attributes (de la Torre, 2009; Rupp & Templin, 2007). Unlike CTT and IRT, which focus on the severity of addiction, CDMs aim to identify the specific components that make up the addiction and determine which aspects are deficient at the individual level. This approach provides a more interpretable method for understanding complex behavioral addictions.

Methodologically, CDMs involve attributes, a Q-matrix, and a classification model. These models probabilistically infer an individual’s mastery of each attribute (Junker & Sijtsma, 2001; Rupp & Templin, 2007), converting assessment results from a single score into a multidimensional attribute profile. This approach aligns well with the diagnostic logic of IGD, as the nine DSM-5 TR IGD criteria have distinct symptom referents that can be defined as binary attributes (mastery/non-mastery), which correspond to clinical decision units for symptoms (American Psychiatric Association, 2022). Attribute-based diagnostic outputs have been shown to offer greater interpretative and intervention relevance in psychological and educational assessments (Ravand & Baghaei, 2020; Tu et al., 2017).

Moreover, CDMs differ fundamentally in their assumptions about how attributes combine to produce a diagnostic response. Noncompensatory models such as DINA assume that all required attributes must co-occur, while compensatory models such as DINO assume that any single attribute is sufficient, and additive models such as A-CDM assume that each attribute contributes independently (de la Torre, 2009; J. L. Templin & Henson, 2006). In the context of IGD, these distinctions carry direct clinical implications: the choice of model determines whether the co-occurrence of multiple symptom dimensions is necessary for disorder classification, or whether individual dimensions can independently indicate clinically meaningful risk (Przybylski et al., 2017). As discussed in Section 4.2, the empirically driven model selection capacity of the G-DINA framework makes it particularly well suited to IGD assessment, where different symptom dimensions may operate through qualitatively different interaction mechanisms. By explicitly modeling the logical relationships between symptoms, CDMs enhance the theoretical coherence of diagnostic conclusions (de la Torre, 2009) and shift the focus of assessment from broad abnormality detection to the identification of specific functional deficits, supporting precision and developmentally informed interventions for adolescent IGD (F. Wang et al., 2024; Zhang et al., 2023).

As summarized in Table 2, the comparison of psychometric theories such as CTT, IRT, and CDMs highlights the core differences in their goals, analytical units, and output types. These distinctions are crucial in understanding the shifts from traditional measurement models to more diagnostic-focused approaches like CDMs (Rupp & Templin, 2007).

Beyond these methodological considerations, the applicability of CDMs in clinical and mental health contexts has been increasingly demonstrated in recent years, providing empirical precedents that further justify the present framework. J. L. Templin and Henson (2006) introduced the DINO model specifically for the measurement of psychological disorders, applying it to the diagnosis of pathological gambling using DSM-IV-TR criteria and showing that CDMs can generate attribute-level diagnostic profiles directly aligned with clinical decision criteria. Extending this line of work, de la Torre et al. (2018) applied a general CDM framework to diagnose personality disorders using item scores from a clinical measurement instrument, demonstrating the viability of CDM-based diagnosis across different psychopathological domains. In the domain of internalizing disorders, D. Wang et al. (2019) developed a new CDM-based instrument for depression, showing that attribute-level diagnostic information derived from CDMs offers greater intervention relevance than traditional total scores. More broadly, Mun et al. (2022) provided a tutorial demonstrating that CDMs can characterize co-occurring mental health symptom profiles, including anxiety, depression, hostility, and alcohol-related problems, using existing item-level data, and argued that CDMs capture clinically meaningful presentations that severity-based approaches tend to obscure. Within the specific domain of behavioral addictions most relevant to the present framework, Tu et al. (2017) developed a CDM-based diagnostic classification test for internet addiction grounded in DSM-5 criteria, demonstrating strong sensitivity and specificity and establishing a direct methodological precedent for the proposed CD-CAT approach to IGD assessment.

## 4. From CDMs and CAT to CD-CAT: Technical Foundations and Integration Logic

### 4.1. Suitability of Computerized Adaptive Testing (CAT) for Adolescent IGD Assessment

Implementing IGD assessment in adolescents involves a specific challenge: cognitive load and compliance. Traditional static fixed-form tests are often lengthy and repetitive, which can induce respondent fatigue (e.g., skipping items, early termination, careless responses) and compromise data quality (Egleston et al., 2011; Ghafourifard, 2024; Jeong et al., 2023). Introducing Computerized Adaptive Testing (CAT) into IGD assessment is therefore not only an efficiency improvement but also an adaptation to adolescents’ cognitive and motivational characteristics.

CAT’s core principle is “testing tailored to the individual.” Unlike fixed-form tests, CAT is a dynamic interactive process (Chang, 2015; Meijer & Nering, 1999). In brief, an item of appropriate difficulty is selected based on an initial estimate; following each response, the latent trait estimate is updated using Bayesian or maximum-likelihood methods, and the next item providing maximum information at the updated estimate is then selected. This cycle continues until the standard error falls below a preset threshold or a maximum number of items is reached.

In IGD assessment, CAT helps to address fatigue and attentional limitations. Adolescents typically have shorter attention spans compared to adults (Hoyer et al., 2021) and lower tolerance for repetitive questionnaire tasks (Mathiowetz & Dipko, 2000). CAT can reduce the length of the test by more than 50% (Paap et al., 2019; Loe et al., 2017), allowing for high-precision measurements within the time frame when adolescents are most focused. This means that CAT has been shown to reduce test length by more than 50% without sacrificing precision (Paap et al., 2019; Loe et al., 2017), allowing accurate assessment within the attentional window when adolescents are most engaged. Furthermore, CAT helps to reduce defensiveness and social desirability bias. In gaming addiction assessments, adolescents may hide certain behaviors to avoid blame and provide defensive responses. Fixed item order can make the purpose of the questions more predictable, which increases the likelihood of socially desirable responses (Stefkovics & Kmetty, 2022). With randomized and adaptive item presentation, anticipatory strategies are disrupted, reducing order-related bias and improving the authenticity of responses (Şahin, 2021).

### 4.2. Integration Logic and Model Selection for CD-CAT

CAT alone improves efficiency, but if it remains grounded in the IRT “total score/θ” logic, it cannot address the diagnostic challenge posed by IGD heterogeneity (Lim & Bangeranye, 2024). Sorrel et al. (2020) noted that combining cognitive diagnosis with CAT enables dynamic item selection to precisely identify complex attribute patterns, compensating for the limited clinical interpretability of unidimensional CAT. CDMs’ diagnostic precision must therefore be integrated with CAT’s efficiency to form CD-CAT. The goal of CD-CAT is to classify individuals into one of 2K possible attribute mastery patterns, where K is the number of attributes. While IRT-based CAT aims to locate a single latent trait θ, CD-CAT requires tailored algorithmic adjustments in three respects.

First, CD-CAT distinguishes same-score, different-structure profiles from a multidimensional perspective. Traditional IRT-CAT selects items along a single severity continuum, whereas CD-CAT selects items to discriminate among attribute patterns. For two adolescents who both appear moderately at risk, CD-CAT can identify whether one primarily reflects an emotion-escape subtype and the other an impulse-loss-of-control subtype, and then deliver targeted items to verify these hypotheses. This profile-based selection allows the system to efficiently differentiate among clinically meaningful subtypes (Cheng, 2009).

Second, CD-CAT simulates clinical reasoning through its item selection strategies. The CD-CAT engine seeks to maximally reduce uncertainty about attribute patterns, using metrics such as Shannon entropy or Kullback–Leibler (KL) information. This is mathematically isomorphic to Bayesian clinical reasoning: rather than asking all questions mechanically, the system updates hypotheses in real time based on each response, prioritizing items that target the most diagnostically discriminating attributes at each step (Yang et al., 2020).

Third, CD-CAT balances efficiency and diagnostic depth through its termination rules. A uniform prior distribution is typically assumed at the outset, assigning equal probability to all permissible attribute patterns; where attribute hierarchies are specified, logically implausible patterns receive a prior of zero (Huebner & Wang, 2011; J. Templin & Bradshaw, 2014). After each response, posterior probabilities are updated via Bayes’ theorem, with MAP or EAP estimation serving as the running classification (Cheng, 2009; Rupp et al., 2010). Variable-length stopping rules, more appropriate for the present framework than fixed-length alternatives, terminate the test once the posterior probability of the modal attribute pattern exceeds a preset threshold (e.g., 0.80 or 0.90). When multiple patterns carry similar probabilities and none reaches the threshold, entropy-or KL-based criteria continue to guide item selection toward maximally discriminating items until the threshold or item limit is met (Cheng, 2009; Yang et al., 2020).

### 4.3. CDM Model Selection for IGD Assessment

A further consideration in implementing CD-CAT for IGD concerns the selection of an appropriate CDM, as different models embody fundamentally different assumptions about attribute relationships that have direct implications for classification outcomes and the interpretation of diagnostic profiles. In the G-DINA framework, different reduced models are derived by imposing specific constraints on the saturated model parameters (de la Torre, 2011). When all lower-order effects are constrained to zero and only the highest-order interaction is retained, the G-DINA model reduces to the noncompensatory DINA model, in which all required attributes must co-occur for a positive response (de la Torre, 2009; Junker & Sijtsma, 2001). When a specific pattern of interaction constraints is applied, the compensatory DINO model is obtained, in which mastery of any single required attribute is sufficient for endorsement (J. L. Templin & Henson, 2006). When all interaction effects are constrained to zero and only additive main effects are retained, the G-DINA model yields the A-CDM and its variants (LLM, R-RUM), in which each attribute contributes independently to the probability of endorsement (de la Torre, 2011).

For IGD assessment specifically, neither a purely noncompensatory nor a purely compensatory model appears theoretically adequate. The DINA model’s requirement that all relevant attributes co-occur would imply that the absence of any single symptom dimension is sufficient to preclude a disorder classification—an assumption that is inconsistent with the DSM-5 TR’s flexible five-of-nine threshold and with the documented heterogeneity of IGD presentations across subtypes (Kökönyei et al., 2019; Zhang et al., 2023). Conversely, the DINO model’s assumption that any single attribute is sufficient for endorsement risks oversimplifying the disorder’s conjunctive diagnostic logic and may lead to overclassification in clinical screening contexts (Przybylski et al., 2017). Furthermore, existing factor-analytic research on the DSM-5 TR IGD criteria reveals a pattern that is difficult to reconcile with any single reduced model: while most studies support a unidimensional overall structure (Király et al., 2017; Pontes & Griffiths, 2015; Schivinski et al., 2018), evidence of multidimensional substructures has also been reported (Pontes et al., 2014; Poon et al., 2021), suggesting that individual symptom criteria may cluster into distinguishable dimensions operating through different interaction mechanisms. The G-DINA model is therefore theoretically most appropriate at the current conceptual stage, as its item-level flexibility allows distinct reduced models, whether noncompensatory, compensatory, or additive, to be applied to different symptom dimensions within a single analysis, with the most appropriate model for each item determined empirically rather than imposed a priori (de la Torre, 2011; Lim & Bangeranye, 2024).

Taken together, these algorithmic and model-theoretic considerations underscore why CD-CAT represents more than a technical combination of CDM and CAT. The clinical characteristics of IGD—its multidimensionality, its symptom heterogeneity, and the adolescent population’s comparatively lower tolerance for lengthy assessments—align precisely with the strengths that CD-CAT brings through algorithmic coordination: standardizing and operationalizing expert-level clinical reasoning in a form that is both efficient and scalable. It is also worth noting that polytomous CD-CAT models for Likert-type responses have been developed, which may further enhance the granularity of IGD symptom assessment beyond binary endorsement (Gao et al., 2020).

## 5. Core Components of the CD-CAT Conceptual Framework

Building on the theoretical rationale established in the preceding sections, this section specifies the core components of the CD-CAT conceptual framework for adolescent IGD assessment. Rather than presenting an implemented system, the framework defines the key theoretical constructs, structural relationships, and methodological logic that must be established before empirical development can proceed.

### 5.1. Attribute Definition: Operationalizing the DSM-5 TR Criteria

Attribute definition is the cornerstone of CD-CAT, as the validity of the entire diagnostic framework depends on whether the chosen clinical criteria can be meaningfully operationalized as discrete, measurable attributes. In this framework, the nine DSM-5 TR IGD criteria are directly operationalized as nine binary attributes, each representing the presence or absence of a clinically distinguishable symptom dimension. The empirical basis for this operationalization is well-established. Each criterion refers to a behaviorally specific and clinically distinguishable symptom domain, and confirmatory factor analyses have supported the structural validity of the nine-criterion model across multiple independent samples (Pontes et al., 2014; Király et al., 2017). IRT analyses have further confirmed that each criterion carries independent diagnostic information with adequate to high discrimination parameters (Schivinski et al., 2018), and cross-cultural measurement invariance has been demonstrated across adolescent samples from multiple countries (Poon et al., 2021). These properties collectively establish a scientifically defensible basis for treating the nine DSM-5 TR criteria as nine distinct, measurable attributes within the proposed framework. The attributes are defined as follows:

A1 Preoccupation: Preoccupation with internet games (e.g., thinking about past gaming activity or anticipating the next game).

A2 Withdrawal: Withdrawal symptoms when gaming is taken away (e.g., irritability, anxiety, sadness).

A3 Tolerance: The need to spend increasing amounts of time gaming or to seek more stimulating games to achieve satisfaction.

A4 Loss of control: Unsuccessful attempts to control, reduce, or stop gaming.

A5 Loss of interest: Loss of interest in previous hobbies and entertainment as a result of, and with the exception of, gaming.

A6 Continued use despite problems: Continued excessive use despite knowledge of psychosocial or physical problems.

A7 Deception: Deceiving family members, therapists, or others regarding the amount of gaming.

A8 Escape: Using gaming to escape or relieve negative moods or real-life stress.

A9 Jeopardizing relationships/opportunities: Jeopardizing or losing a significant relationship, job, or educational/career opportunity because of gaming.

### 5.2. Q-Matrix Construction and Validation Logic

The Q-matrix is a critical component of the CD-CAT framework, serving as the blueprint that formally specifies the relationship between items and the attributes they are designed to measure. Building on the attribute system defined in Section 5.1, Q-matrix construction for IGD assessment involves four interrelated stages.

The first stage involves item sourcing and content-based attribute mapping. Item sources for the IGD item bank can follow two complementary pathways: adapting existing validated items from DSM-5 TR-based instruments such as the IGDS9-SF and IGDT-10, which provide a proven content validity foundation; and developing new items by clinical experts to cover symptom expressions that are underrepresented in existing tools. Once items are assembled, domain experts independently evaluate which DSM-5 TR criterion each item primarily measures and assign the corresponding attribute code. For example, the item “Do you lie to your parents about how much time you spend gaming?” is mapped to A7 (Deception). Items must be reviewed for clarity, developmental appropriateness for adolescents, and freedom from social desirability bias, given that adolescents may be particularly prone to minimizing gaming-related difficulties (Stefkovics & Kmetty, 2022).

The second stage addresses multi-attribute item design. Clinically, IGD symptoms frequently co-occur, and certain items are designed to capture this co-occurrence. For example, the item “Even though I know gaming affects my studies, I keep playing” simultaneously reflects A6 (Continued use despite problems) and A9 (Jeopardizing relationships/opportunities), as persistent gaming despite academic consequences involves both ongoing use and the sacrifice of important life domains. Multi-attribute items are not only theoretically permissible but also practically necessary for capturing the complex, overlapping symptom structure of IGD (Rupp & Templin, 2007). This also explains why the number of items in a Q-matrix need not equal the number of attributes, as illustrated in Table 3 below, where Item 10 involves both A2 (Withdrawal) and A4 (Loss of control).

The third stage involves hierarchical structure analysis among IGD attributes. The nine DSM-5 TR criteria are not necessarily independent; some may have theoretically grounded prerequisite relationships that reflect the clinical progression of IGD. For instance, A3 (Tolerance) may constitute a physiological precursor to A2 (Withdrawal), such that “Withdrawal present, Tolerance absent” is clinically implausible. Similarly, A4 (Loss of control) may function as a prerequisite for A9 (Jeopardizing relationships/opportunities), as significant real-life consequences are unlikely without prior loss of behavioral control. Identifying such hierarchies allows logically implausible attribute patterns to be excluded from the prior distribution, reducing the classification search space and improving diagnostic efficiency (Y. Cai et al., 2018).

The fourth stage involves data-driven Q-matrix validation and iterative refinement. Given that inter-expert disagreements are common in IGD criterion interpretation—for example, whether a specific item reflects “escape” or “loss of control” depends partly on clinical judgment—expert-based specification alone cannot guarantee Q-matrix accuracy. Statistical methods such as the EM-based Q-matrix refinement algorithm can identify discrepancies between the theoretically specified Q-matrix and empirical response patterns, enabling iterative correction (F. Wang et al., 2024). A misspecified Q-matrix can propagate systematic classification errors across the entire item bank, making this validation stage critical to the integrity of the IGD diagnostic framework. In addition to Q-matrix misspecification detection, model-data fit should be evaluated using both absolute and relative fit indices. Absolute fit indices such as the M2 statistic and its associated RMSEA_2_ assess the overall discrepancy between model-implied and observed response patterns, while relative fit indices such as AIC and BIC enable comparison among competing CDMs (e.g., DINA, DINO, G-DINA) to inform model selection (Chen et al., 2013; Liu et al., 2016). Together, these methods provide a comprehensive empirical basis for evaluating and refining the IGD diagnostic framework.

Table 3 below provides a partial, illustrative example of a Q-matrix for adolescent IGD cognitive diagnosis, demonstrating the structural logic of attribute-item mapping. In practical applications, a full Q-matrix would require items numbering in the hundreds, with sufficient coverage across all nine attributes and varying levels of clinical severity to ensure reliable classification.

### 5.3. Adaptive Diagnostic Process: Conceptual Workflow

The five-stage conceptual workflow outlined below describes how the algorithmic mechanisms specified in Section 4.2, including prior distribution setting, Bayesian posterior updating, entropy- or KL-based item selection, and variable-length stopping rules, would operate in practice to guide the diagnostic process from initial screening to final classification.

Stage 1. Initial probing. The diagnostic process begins with broadly informative items designed to provide an initial estimate of the respondent’s attribute profile. For example, an item probing A1 (Preoccupation) serves as a low-threshold entry point that is likely to be informative across a wide range of attribute patterns, establishing a starting point for subsequent hypothesis generation.

Stage 2. Dynamic hypothesis generation and posterior updating. Following each item response, the posterior probability distribution over all 2^9^ possible attribute patterns is updated via Bayes’ theorem. An affirmative response to the initial item increases the posterior probability of patterns containing A1, directing subsequent item selection toward attributes that most effectively discriminate among the highest-probability competing patterns.

Stage 3. Targeted follow-up. As the posterior distribution becomes more concentrated, the item selection algorithm, guided by Shannon entropy or KL information criteria, identifies the attributes that remain most uncertain and prioritizes items that target those attributes. For instance, if A4 (Loss of control) and A8 (Escape) emerge as the key discriminators among the leading competing patterns, items measuring these attributes receive the highest selection priority.

Stage 4. Elimination-based narrowing. Negative responses to items associated with specific attributes progressively reduce the posterior probability of patterns containing those attributes, narrowing the effective search space. For example, consistent denial of A2 (Withdrawal) and A7 (Deception) items would substantially down-weight patterns in which these attributes are present, converging the posterior distribution toward patterns consistent with the observed response data.

Stage 5. Convergence and output. The adaptive process terminates once the posterior probability of the modal attribute pattern exceeds the preset stopping threshold (e.g., 0.80 or 0.90), typically within approximately 10–15 items depending on the complexity of the individual’s symptom profile. The resulting attribute mastery pattern—for example, endorsement of A1, A3, A4, and A8 but not others—is then passed to the output module described in Section 5.4, which generates the structured diagnostic report.

### 5.4. Diagnostic Output and Intervention Linkage

The diagnostic output of the proposed CD-CAT framework is not a single numerical score, but a structured, multi-component report designed to support individualized clinical interpretation and intervention planning. Three interrelated output components are specified.

The first component is an attribute profile visualization, which presents the individual’s endorsement status across all nine DSM-5 TR attributes in a graphical format such as a radar or bar chart. Unlike a total score, which conveys only the overall severity of IGD, the attribute profile makes the internal symptom structure directly visible, distinguishing, for instance, a profile dominated by emotion-regulation difficulties (high A2 Withdrawal, A8 Escape) from one characterized primarily by behavioral dyscontrol (high A4 Loss of control, A6 Continued use despite problems). This visual representation supports more nuanced clinical communication with adolescents, parents, and educators, and is consistent with the broader case for attribute-based assessment in clinical contexts (Mun et al., 2022).

The second component is a diagnostic classification, which maps the endorsed attribute pattern onto the DSM-5 TR diagnostic threshold. If five or more attributes are endorsed, the output specifies an IGD-positive classification and identifies which specific criteria are met, providing a direct link between the psychometric output and the clinical diagnostic framework. For cases falling below the diagnostic threshold, the profile still provides clinically actionable information by identifying subclinical symptom dimensions that may warrant monitoring or early intervention, thereby supporting preventive as well as diagnostic applications.

The third component is an intervention linkage, which translates the attribute profile into targeted clinical recommendations. Because different attribute configurations correspond to different pathological subtypes, the recommendations generated are profile-specific rather than generic. For example, profiles in which A8 (Escape) and A5 (Loss of interest) are prominent suggest emotion-regulation and behavioral activation strategies such as reconnecting with real-life activities and developing alternative leisure interests; profiles in which A4 (Loss of control) is prominent suggest impulse control training as part of a cognitive-behavioral approach (G. H. Dong et al., 2024). This direct mapping from diagnostic profile to intervention target closes the assessment-intervention loop that traditional total-score tools leave open, and is consistent with the clinical rationale for CDM-based assessment demonstrated by Tu et al. (2017).

Together, these three output components reflect the core clinical promise of the proposed framework: to move IGD assessment beyond the question of ‘how severe’ toward the more actionable question of ‘which specific symptom dimensions are present and what should be done about them.’ The conceptual output structure described here is intended as a specification for future instrument development rather than a description of an existing report format.

## 6. Discussion

### 6.1. Theoretical Contributions

The CD-CAT conceptual assessment framework proposed in this study advances the field of adolescent IGD assessment in three interconnected ways.

First, the framework reconceptualizes IGD assessment by operationalizing the nine DSM-5 TR criteria as discrete binary attributes within a Q-matrix structure, establishing a theoretically coherent mapping between clinical diagnostic categories and psychometric measurement. Existing CTT- and IRT-based assessment tools characterize IGD along a single severity continuum, a structure that is ill-suited to capturing the disorder’s documented symptom heterogeneity and subtype variation (Kökönyei et al., 2019; Zhang et al., 2023). CDM-based attribute profiling, by contrast, produces a diagnostic output that is structurally aligned with the clinical decision units embedded in the DSM-5 TR criteria, offering a more interpretable basis for individualized assessment (de la Torre, 2009; Rupp et al., 2010).

Second, the integration of CDMs with CAT addresses a recognized limitation of each approach when applied independently. CDMs generate fine-grained diagnostic profiles but require fixed item sets, which limit their efficiency in large-scale screening contexts. CAT improves measurement efficiency through adaptive item selection but, when grounded solely in IRT, remains constrained to estimating a single latent severity dimension. The CD-CAT combination enables attribute-targeted adaptive item selection, allowing the system to efficiently discriminate among clinically meaningful symptom patterns rather than positioning individuals on a unidimensional scale (Cheng, 2009; Sorrel et al., 2020).

Third, by grounding the attribute system in the DSM-5 TR criteria whose psychometric properties have been substantiated across multiple independent samples (Schivinski et al., 2018; Király et al., 2017), the framework ensures that its diagnostic structure is empirically defensible. The explicit specification of Q-matrix construction logic, adaptive item selection procedures, and profile-based output further provides a structured foundation upon which future empirical development can be systematically built (Tu et al., 2017).

### 6.2. Limitations

The limitations of the present study are inherent to its status as a conceptual assessment framework paper. The proposed CD-CAT framework is grounded in theoretical reasoning and has not been subjected to empirical validation of any form, including simulation studies, pilot testing, item calibration, or classification accuracy evaluation. While the development of a theoretically specified conceptual framework constitutes a recognized and necessary contribution to the advancement of assessment methodology (Rupp et al., 2010), the empirical properties of the proposed attribute system, Q-matrix structure, and adaptive item selection logic remain to be established in future research.

Two content-level limitations are also acknowledged. First, the framework adopts the nine DSM-5 TR criteria as the basis for attribute definition, treating them as equally discrete and mutually independent diagnostic dimensions. However, existing factor-analytic and IRT evidence presents a more nuanced picture: while most studies support a unidimensional overall structure for the nine criteria (Király et al., 2017; Pontes & Griffiths, 2015; Schivinski et al., 2018), evidence of multidimensional substructures has also been reported (Pontes et al., 2014; Poon et al., 2021), and the diagnostic utility of individual criteria has been shown to vary across symptom dimensions and cultural contexts (Luo et al., 2022; Borges et al., 2021). These findings suggest that the equal-weighting assumption embedded in the current one-to-one attribute mapping warrants careful empirical scrutiny. Second, the expert-based attribute-item mapping process introduces a degree of subjectivity that data-driven validation methods can partially address but cannot fully resolve without empirical testing (de la Torre, 2008; F. Wang et al., 2024). Alternative attribute assignments of comparable theoretical plausibility cannot be excluded, and the extent to which the proposed mappings accurately reflect the underlying symptom structure of adolescent IGD remains an open empirical question.

### 6.3. Future Directions

Advancing the CD-CAT framework from its current conceptual stage to empirical application will require systematic research addressing its psychometric foundations, model specifications, and cross-cultural generalizability. Priorities include Q-matrix validation, CDM model selection and fit evaluation, and item parameter calibration across representative adolescent samples. Extending the framework to accommodate ICD-11-based diagnostic contexts and examining its measurement properties across culturally diverse populations represent additional directions that would strengthen the framework’s scientific foundation and broaden its clinical applicability.

## 7. Conclusions

This study proposes CD-CAT as a conceptual assessment framework for adolescent IGD assessment, integrating the multidimensional diagnostic precision of Cognitive Diagnostic Models with the measurement efficiency of Computerized Adaptive Testing. Grounded in the nine DSM-5 TR criteria as operationalized diagnostic attributes, the framework offers a theoretically coherent structure that bridges clinical diagnostic logic and psychometric modeling, a gap that existing unidimensional assessment approaches have not addressed. By specifying the logic of Q-matrix construction, adaptive item selection, and profile-based diagnostic output, this work provides a structured foundation for future empirical development. It is our hope that this conceptual contribution will stimulate systematic empirical investigation and ultimately support the development of more precise, efficient, and clinically meaningful assessment tools for adolescent IGD.

## Figures and Tables

**Table 1:** Comparison of Diagnostic Criteria for Gaming-Related Disorders in DSM-5 and ICD-11.

Feature	DSM-5 TR	ICD-11
Official Name	Internet Gaming Disorder, IGD	Gaming Disorder, GD
Core Definition	The essential feature of Internet gaming disorder is a pattern of excessive and prolonged participation in Internet gaming that results in a cluster of cognitive and behavioral symptoms, including progressive loss of control over gaming, tolerance, and withdrawal.	Gaming disorder is characterised by a pattern of persistent or recurrent gaming behaviour (‘digital gaming’ or ‘video-gaming’), which may be online or offline.
Specific Criteria	Preoccupation with Internet games. Withdrawal symptoms when Internet gaming is taken away. Tolerance—the need to spend increasing amounts of time engaged in Internet games. Unsuccessful attempts to control participation in Internet games. Loss of interest in previous hobbies and entertainment as a result of, and with the exception of, Internet games. Continued excessive use of Internet games despite knowledge of psychosocial problems. Has deceived family members, therapists, or others regarding the amount of Internet gaming. Use of Internet games to escape or relieve a negative mood. Has jeopardized or lost a significant relationship, job, or educational or career opportunity because of participation in Internet games.	Impaired control over gaming behaviour (e.g., onset, frequency, intensity, duration, termination, context). Increasing priority given to gaming behaviour to the extent that gaming takes precedence over other life interests and daily activities. Continuation or escalation of gaming behaviour despite negative consequences (e.g., family conflict due to gaming behaviour, poor scholastic performance, negative impact on health).
Diagnostic Threshold	Meets at least five of the nine criteria in a 12-month period	The gaming behaviour and other features are normally evident over a period of at least 12 months in order for a diagnosis to be assigned, although the required duration may be shortened if all diagnostic requirements are met and symptoms are severe.
Classification	Mild/Moderate/Severe (depending on the degree of disruption of normal activities)	Met criteria/Did not meet criteria

**Table 2:** The Comparative Analysis of Psychometric Theories: CTT, IRT, and CDMs (Rupp & Templin, 2007).

Dimension	Classical Test Theory (CTT)	Item Response Theory (IRT)	Cognitive Diagnostic Models (CDMs)
Primary objective	Assessing the reliability and validity of total scores, estimating an individual’s true score	Estimating an individual’s level on a continuous latent trait, evaluating item parameters	Classifying an individual’s mastery of a set of discrete attributes, providing a diagnostic profile
Unit of analysis	Entire test	Individual items	Relationship between items and attributes
Type of latent variable	Continuous (implicitly assumed)	Continuous	Discrete (categorical)
Key parameters	Item difficulty (proportion correct), discrimination (point-biserial correlation)	Item difficulty, discrimination, guessing	Item slip and guessing parameters; Q-matrix
Sample dependence	Sample dependent	Sample invariant	Sample invariant
Typical output	A single total score (raw or standardized) and test-level reliability estimates	Ability estimate (θ) on a latent continuum with standard error	Attribute mastery/non-mastery profiles with posterior probabilities

**Table 3:** Example Q-Matrix for Adolescent IGD Cognitive Diagnosis (Partial).

Items (Example)	A1	A2	A3	A4	A5	A6	A7	A8	A9
1. I rarely think about anything other than gaming.	1	0	0	0	0	0	0	0	0
2. If I do not play for a few days, I feel very uncomfortable.	0	1	0	0	0	0	0	0	0
3. I need to play for longer to feel satisfied.	0	0	1	0	0	0	0	0	0
4. I have tried many times to play less but failed.	0	0	0	1	0	0	0	0	0
5. I lost interest in activities I used to enjoy (e.g., sports, socializing).	0	0	0	0	1	0	0	0	0
6. Even though I know gaming affects my studies, I keep playing.	0	0	0	0	0	1	0	0	1
7. I hide my actual gaming time from my family.	0	0	0	0	0	0	1	0	0
8. I play games to forget worries or unhappiness.	0	0	0	0	0	0	0	1	0
9. I argue with my parents because of gaming.	0	0	0	0	0	0	0	0	1
10. I tried to reduce gaming, but I felt anxious as soon as I stopped.	0	1	0	1	0	0	0	0	0
(more items) …									

## Data Availability

No new data were created or analyzed in this study. Data sharing is not applicable to this article.

## References

[B1-behavsci-16-00558] Ahmed M. Z., Ahmed O., Gao L., Jobe M. C., Li W. (2024). Internet gaming disorder and mental health of children in China: A latent profile analysis. International Journal of Mental Health Promotion.

[B2-behavsci-16-00558] Allen M. J., Yen W. M. (2001). Introduction to measurement theory.

[B3-behavsci-16-00558] American Psychiatric Association (2022). Diagnostic and statistical manual of mental disorders.

[B4-behavsci-16-00558] Borges G., Orozco R., Benjet C., Martinez K. I. M., Contreras E. V., Perez A. L. J. E., Cedr Es A. J. P. A., Uribe P. C. H. A., Couder M. I. A. C. D. I., Gutierrez-Garcia R. U. A., Chavez G. E. Q., Albor Y., Mendez E., Medina-Mora M. E., Mortier P., Ayuso-Mateos J. E. L. (2021). (Internet) Gaming disorder in DSM-5 and ICD-11: A case of the glass half empty or half full. Canadian Journal of Psychiatry.

[B5-behavsci-16-00558] Cai L., Choi K., Hansen M., Harrell L. (2016). Item response theory. Annual Review of Statistics and Its Application.

[B6-behavsci-16-00558] Cai Y., Tu D., Ding S. (2018). Theorems and methods of a complete Q matrix with attribute hierarchies under restricted Q-matrix design. Frontiers in Psychology.

[B7-behavsci-16-00558] Chang H. H. (2015). Psychometrics behind computerized adaptive testing. Psychometrika.

[B8-behavsci-16-00558] Chen J., de la Torre J., Zhang Z. (2013). Relative and absolute fit evaluation in cognitive diagnosis modeling. Journal of Educational Measurement.

[B9-behavsci-16-00558] Cheng Y. (2009). When cognitive diagnosis meets computerized adaptive testing: CD-CAT. Psychometrika.

[B10-behavsci-16-00558] Cudo A., Starzak P., Szubielska M. (2024). The relationship between gaming disorder, frequency of playing action games, game context, and cognitive control. Advances in Cognitive Psychology.

[B11-behavsci-16-00558] de la Torre J. (2008). An empirically based method of Q-matrix validation for the DINA model: Development and applications. Journal of Educational Measurement.

[B12-behavsci-16-00558] de la Torre J. (2009). A cognitive diagnosis model for cognitively based multiple-choice options. Applied Psychological Measurement.

[B13-behavsci-16-00558] de la Torre J. (2011). The generalized DINA model framework. Psychometrika.

[B14-behavsci-16-00558] de la Torre J., van der Ark L. A., Rossi G. (2018). Analysis of clinical data from a cognitive diagnosis modeling framework. Measurement and Evaluation in Counseling and Development.

[B15-behavsci-16-00558] Dong G. H., Dai J., Potenza M. N. (2024). Ten years of research on the treatments of internet gaming disorder: A scoping review and directions for future research. Journal of Behavioral Addictions.

[B16-behavsci-16-00558] Dong X., Jiang Y., Zhang Y., Zhang W., Wang N. (2022). Hazards of game addiction to health in adolescents. Shanghai Journal of Preventive Medicine.

[B17-behavsci-16-00558] Düll L., Müller A., Steins-Loeber S. (2024). Negative consequences experienced by individuals with gaming disorder symptoms: A systematic review of available longitudinal studies. Current Addiction Reports.

[B18-behavsci-16-00558] Egleston B. L., Miller S. M., Meropol N. J. (2011). The impact of misclassification due to survey response fatigue on estimation and identifiability of treatment effects. Statistics in Medicine.

[B19-behavsci-16-00558] Embretson S. E., Reise S. P. (2013). Item response theory for psychologists.

[B20-behavsci-16-00558] Fam J. Y. (2018). Prevalence of internet gaming disorder in adolescents: A meta-analysis across three decades. Scandinavian Journal of Psychology.

[B21-behavsci-16-00558] Gao X., Wang D., Cai Y., Tu D. (2020). Cognitive diagnostic computerized adaptive testing for polytomously scored items. Journal of Classification.

[B22-behavsci-16-00558] Gentile D. A., Bailey K., Bavelier D., Brockmyer J. F., Cash H., Coyne S. M., Doan A., Grant D. S., Green C. S., Griffiths M., Markle T., Petry N. M., Prot S., Rae C. D., Rehbein F., Rich M., Sullivan D., Woolley E., Young K. (2017). Internet gaming disorder in children and adolescents. Pediatrics.

[B23-behavsci-16-00558] Ghafourifard M. (2024). Survey fatigue in questionnaire based research: The issues and solutions. Journal of Caring Sciences.

[B24-behavsci-16-00558] Gisbert-Pérez J., Longobardi C., Martí-Vilar M., Mastrokoukou S., Badenes-Ribera L. (2025). Prevalence of Internet gaming disorder in young adults: A systematic review and meta-analysis. Addictive Behaviors.

[B25-behavsci-16-00558] Hoyer R. S., Elshafei H., Hemmerlin J., Bouet R., Bidet-Caulet A. (2021). Why are children so distractible? Development of attention and motor control from childhood to adulthood. Child Development.

[B26-behavsci-16-00558] Hu Y. (2022). Connotation, etiology, and intervention of internet gaming addiction. Advances in Psychology.

[B27-behavsci-16-00558] Huebner A., Wang C. (2011). A note on comparing examinee classification methods for cognitive diagnosis models. Educational and Psychological Measurement.

[B28-behavsci-16-00558] Jabrayilov R., Emons W. H., Sijtsma K. (2016). Comparison of classical test theory and item response theory in individual change assessment. Applied Psychological Measurement.

[B29-behavsci-16-00558] Jeong D., Aggarwal S., Robinson J., Kumar N., Spearot A., Park D. S. (2023). Exhaustive or exhausting? Evidence on respondent fatigue in long surveys. Journal of Development Economics.

[B30-behavsci-16-00558] Junker B. W., Sijtsma K. (2001). Cognitive assessment models with few assumptions, and connections with nonparametric item response theory. Applied Psychological Measurement.

[B31-behavsci-16-00558] Király O., Sleczka P., Pontes H. M., Urbán R., Griffiths M. D., Demetrovics Z. (2017). Validation of the ten-item internet gaming disorder test (IGDT-10) and evaluation of the nine DSM-5 Internet gaming disorder criteria. Addictive Behaviors.

[B32-behavsci-16-00558] Kökönyei G., Kocsel N., Király O., Griffiths M. D., Galambos A., Magi A., Paksi B., Demetrovics Z. (2019). The role of cognitive emotion regulation strategies in problem gaming among adolescents: A nationally representative survey study. Frontiers in Psychiatry.

[B33-behavsci-16-00558] Lim Y. S., Bangeranye C. (2024). The choice between cognitive diagnosis and item response theory: A case study from medical education. International Journal of Testing.

[B34-behavsci-16-00558] Liu Y., Tian W., Xin T. (2016). An application of M_2_ statistic to evaluate the fit of cognitive diagnostic models. Journal of Educational and Behavioral Statistics.

[B35-behavsci-16-00558] Loe B. S., Stillwell D., Gibbons C. (2017). Computerized adaptive testing provides reliable and efficient depression measurement using the CES-D scale. Journal of Medical Internet Research.

[B36-behavsci-16-00558] Luo T., Wei D., Guo J., Hu M., Chao X., Sun Y., Sun Q., Xiao S., Liao Y. (2022). Diagnostic contribution of the DSM-5 criteria for internet gaming disorder. Frontiers in Psychiatry.

[B37-behavsci-16-00558] Mathiowetz N. A., Dipko S. M. (2000). A comparison of response error by adolescents and adults: Findings from a health care study. Medical Care.

[B38-behavsci-16-00558] Meijer R. R., Nering M. L. (1999). Computerized adaptive testing: Overview and introduction. Applied Psychological Measurement.

[B39-behavsci-16-00558] Mihara S., Higuchi S. (2017). Cross-sectional and longitudinal epidemiological studies of Internet gaming disorder: A systematic review of the literature. Psychiatry and Clinical Neurosciences.

[B40-behavsci-16-00558] Mun E. Y., de la Torre J., Atkins D. C., White H. R., Ray A. E., Kim S.-Y., Jiao Y., Clarke N., Huo Y. (2022). A tutorial on cognitive diagnosis modeling for characterizing mental health symptom profiles using existing item responses. Prevention Science.

[B41-behavsci-16-00558] Müller K. W., Beutel M. E., Dreier M., Wölfling K. (2019). A clinical evaluation of the DSM-5 criteria for internet gaming disorder and a pilot study on their applicability to further internet-related disorders. Journal of Behavioral Addictions.

[B42-behavsci-16-00558] Paap M. C., Born S., Braeken J. (2019). Measurement efficiency for fixed-precision multidimensional computerized adaptive tests: Comparing health measurement and educational testing using example banks. Applied Psychological Measurement.

[B43-behavsci-16-00558] Paschke K., Austermann M. I., Thomasius R. (2020). Assessing ICD-11 gaming disorder in adolescent gamers: Development and validation of the gaming disorder scale for adolescents (GADIS-A). Journal of Clinical Medicine.

[B44-behavsci-16-00558] Paulus F. W., Ohmann S., von Gontard A., Popow C. (2018). Internet gaming disorder in children and adolescents: A systematic review. Developmental Medicine & Child Neurology.

[B45-behavsci-16-00558] Pontes H. M., Griffiths M. D. (2015). Measuring DSM-5 internet gaming disorder: Development and validation of a short psychometric scale. Computers in Human Behavior.

[B46-behavsci-16-00558] Pontes H. M., Király O., Demetrovics Z., Griffiths M. D. (2014). The conceptualisation and measurement of DSM-5 Internet Gaming Disorder: The development of the IGD-20 Test. PLoS ONE.

[B47-behavsci-16-00558] Poon L. Y., Tsang H. W., Chan T. Y., Man S. W., Ng L. Y., Wong Y. L., Lin C. Y., Chien C. W., Griffiths M. D., Pontes H. M., Pakpour A. H. (2021). Psychometric properties of the internet gaming disorder scale–short-form (IGDS9-SF): Systematic review. Journal of Medical Internet Research.

[B48-behavsci-16-00558] Przybylski A. K., Weinstein N., Murayama K. (2017). Internet gaming disorder: Investigating the clinical relevance of a new phenomenon. American Journal of Psychiatry.

[B49-behavsci-16-00558] Ravand H., Baghaei P. (2020). Diagnostic classification models: Recent developments, practical issues, and prospects. International Journal of Testing.

[B50-behavsci-16-00558] Razum J., Glavak-Tkalić R. (2025). Prevalence and impact of internet gaming disorder: A population-based study. Cyberpsychology: Journal of Psychosocial Research on Cyberspace.

[B51-behavsci-16-00558] Rehbein F., Kliem S., Baier D., Mößle T., Petry N. M. (2015). Prevalence of internet gaming disorder in German adolescents: Diagnostic contribution of the nine DSM-5 criteria in a state-wide representative sample. Addiction.

[B52-behavsci-16-00558] Rupp A. A., Templin J., Henson R. (2010). Diagnostic measurement: Theory, methods, and applications.

[B53-behavsci-16-00558] Rupp A. A., Templin J. L. (2007). Unique characteristics of cognitive diagnosis models. Annual meeting of the national council on measurement in education, Chicago.

[B54-behavsci-16-00558] Satapathy P., Khatib M. N., Balaraman A. K., Kaur M., Srivastava M., Barwal A., Prasad G. V. S., Rajput P., Syed R., Sharma G., Kumar S., Singh M. P., Bushi G., Chilakam N., Pandey S., Brar M., Mehta R., Sah S., Gaidhane A., Samal S. K. (2025). Burden of gaming disorder among adolescents: A systemic review and meta-analysis. Public Health in Practice.

[B55-behavsci-16-00558] Sánchez-Iglesias I., Bernaldo-de-Quirós M., Labrador F. J., Estupiñá Puig F. J., Labrador M., Fernández-Arias I. (2020). Spanish validation and scoring of the internet gaming disorder scale-short form (IGDS9-SF). The Spanish Journal of Psychology.

[B56-behavsci-16-00558] Schivinski B., Brzozowska-Woś M., Buchanan E. M., Griffiths M. D., Pontes H. M. (2018). Psychometric assessment of the internet gaming disorder diagnostic criteria: An item response theory study. Addictive Behaviors Reports.

[B57-behavsci-16-00558] Sorrel M. A., Barrada J. R., de la Torre J., Abad F. J. (2020). Adapting cognitive diagnosis computerized adaptive testing item selection rules to traditional item response theory. PLoS ONE.

[B58-behavsci-16-00558] Stefkovics Á., Kmetty Z. (2022). A comparison of question order effects on item-by-item and grid formats: Visual layout matters. Measurement Instruments for the Social Sciences.

[B59-behavsci-16-00558] Şahin M. D. (2021). Effect of item order on certain psychometric properties: A demonstration on a cyberloafing scale. Frontiers in Psychology.

[B60-behavsci-16-00558] Templin J., Bradshaw L. (2014). Hierarchical diagnostic classification models: A family of models for estimating and testing attribute hierarchies. Psychometrika.

[B61-behavsci-16-00558] Templin J. L., Henson R. A. (2006). Measurement of psychological disorders using cognitive diagnosis models. Psychological Methods.

[B62-behavsci-16-00558] Thomas M. L. (2019). Advances in applications of item response theory to clinical assessment. Psychological Assessment.

[B63-behavsci-16-00558] Thompson N. (2023). Classical test theory vs. item response theory.

[B64-behavsci-16-00558] Tse N., Pang N. S. N., Wang X., Li Y., Lo C. K. M., Yang X. (2025). The roles of binge gaming in social, academic and mental health outcomes and gender differences: A school-based survey in Hong Kong. PLoS ONE.

[B65-behavsci-16-00558] Tu D., Gao X., Wang D., Cai Y. (2017). A new measurement of internet addiction using diagnostic classification models. Frontiers in Psychology.

[B66-behavsci-16-00558] Wang D., Gao X., Cai Y., Tu D. (2019). Development of a new instrument for depression with cognitive diagnosis models. Frontiers in Psychology.

[B67-behavsci-16-00558] Wang F., Gao W., Liu Q., Li J., Zhao G., Zhang Z., Huang Z., Zhu M., Wang S., Tong W., Chen E. (2024). A survey of models for cognitive diagnosis: New developments and future directions. arXiv.

[B68-behavsci-16-00558] World Health Organization (2019). International classification of diseases for mortality and morbidity statistics.

[B69-behavsci-16-00558] Yang J., Chang H. H., Tao J., Shi N. (2020). Stratified item selection methods in cognitive diagnosis computerized adaptive testing. Applied Psychological Measurement.

[B70-behavsci-16-00558] Zhang L., Liu M., Yuan M., Hou M., Yang C., Wang Y., Hao W., Liao Y. (2023). The latent profile analysis of Chinese adolescents’ gaming disorder: Examination and validation. BMC Psychiatry.

[B71-behavsci-16-00558] Zhang L., Luo T., Hao W., Cao Y., Yuan M., Liao Y. (2022). Gaming disorder symptom questionnaire: The development and validation of a screening tool for ICD-11 gaming disorder in adolescents. Frontiers in Psychiatry.

[B72-behavsci-16-00558] Zhong N., Du J., Poznyak V., Zhao M., Hao W. (2018). Research progress on gaming disorder and controversies as a new diagnostic category for mental and behavioral disorders in ICD-11 (draft). Chinese Journal of Psychiatry.

